# Growth of Highly c-Axis Oriented AlScN Films on Commercial Substrates

**DOI:** 10.3390/mi13050783

**Published:** 2022-05-17

**Authors:** Jingxiang Su, Simon Fichtner, Muhammad Zubair Ghori, Niklas Wolff, Md. Redwanul Islam, Andriy Lotnyk, Dirk Kaden, Florian Niekiel, Lorenz Kienle, Bernhard Wagner, Fabian Lofink

**Affiliations:** 1 Fraunhofer Institute for Silicon Technology ISIT, Fraunhoferstrasse 1, 25524 Itzehoe, Germany; simon.fichtner@isit.fraunhofer.de (S.F.); muhammad.zubair.ghori@isit.fraunhofer.de (M.Z.G.); dirk.kaden@isit.fraunhofer.de (D.K.); florian.niekiel@isit.fraunhofer.de (F.N.); beha.wagner@gmail.com (B.W.); 2 Institute for Material Science, Kiel University, Kaiserstr. 2, 24143 Kiel, Germany; niwo@tf.uni-kiel.de (N.W.); mdis@tf.uni-kiel.de (M.R.I.); lk@tf.uni-kiel.de (L.K.); 3 Leibniz Institute of Surface Engineering (IOM), Permoserstr. 15, D-04318 Leipzig, Germany; andriy.lotnyk@iom-leipzig.de

**Keywords:** aluminium scandium nitride, piezoelectric thin films, MEMS, non-metallic substrates

## Abstract

In this work, we present a method for growing highly *c*-axis oriented aluminum scandium nitride (AlScN) thin films on (100) silicon (Si), silicon dioxide (SiO_2_) and epitaxial polysilicon (poly-Si) substrates using a substrate independent approach. The presented method offers great advantages in applications such as piezoelectric thin-film-based surface acoustic wave devices where a metallic seed layer cannot be used. The approach relies on a thin AlN layer to establish a wurtzite nucleation layer for the growth of *w*-AlScN films. Both AlScN thin film and seed layer AlN are prepared by DC reactive magnetron sputtering process where a Sc concentration of 27% is used throughout this study. The crystal quality of (0002) orientation of Al_0.73_Sc_0.27_N films on all three substrates is significantly improved by introducing a 20 nm AlN seed layer. Although AlN has a smaller capacitance than AlScN, limiting the charge stored on the electrode plates, the combined piezoelectric coefficient d_33,f_ with 500 nm AlScN is only slightly reduced by about 4.5% in the presence of the seed layer.

## 1. Introduction

Already for decades, piezoelectric thin film AlN has been of interest for its excellent dielectric properties as well as its chemical and temperature stability and has been widely used in piezoelectric MEMS (microelectromechanical systems) sensors and actuator elements [1,2,3,4]. In 2009, Akiyama et al. first reported that the piezoelectric coefficient of AlN could be significantly increased by doping with Sc [5,6]. Since then, AlScN has attracted great attention and has become a promising piezoelectric material for MEMS applications [7,8,9,10,11]. Multiple studies on AlScN-based MEMS magnetroelectric sensors [12], MEMS energy harvesters [13], MEMS quasistatic mirrors [14], and acoustic wave resonators [15,16,17,18,19,20,21] have been reported.

In most piezoelectric MEMS devices, AlN or AlScN thin films are grown on metallic seed layers such as molybdenum (Mo) or platinum (Pt) to ensure good *c*-axis orientation, where full width at half maximum (FWHM) values of the rocking curve measurements are typically larger than 1.3${}^{\circ }$ for AlN and larger than 1.6${}^{\circ }$ for Al_0.73_Sc_0.27_N [22,23,24,25,26]. However, metallic seed layers cannot be used for, e.g., piezoelectric thin-film-based surface acoustic wave (SAW) devices [21], optical waveguides [27], or MEMS actuators with doped silicon used as bottom electrode [28]. For these applications, the piezoelectric layer (AlN or AlScN) has to be grown directly on substrates such as (100) silicon (Si), silicon dioxide (SiO_2_) or epitaxial polysilicon (poly-Si), but still a high degree of *c*-axis orientation of AlN or AlScN is required. For AlN the deposition on various Si-based substrates has been studied by Jiao et al. in [29] and SiO_2_ was found to be most suitable substrate for AlN*c*-axis growth. Due to its higher piezoelectric response compared to AlN, AlScN films have attracted more interest, thus becoming a focus in piezoelectric MEMS research. Although there is success in growing AlScN films with low Sc concentrations on different nonmetallic substrates (on high-resistivity (100) Si [21], low-resistivity boron-doped (001) Si [17,30], SiC [31]), higher Sc concentrations present an increasingly difficult challenge for the growth of AlScN films with exclusive *c*-axis orientation [10].

In this work, we present a largely substrate-independent method to grow wurtzite-type AlScN films with exclusive *c*-axis orientation even for high Sc concentrations. Throughout this paper, AlScN with 27% Sc is chosen as a balance between high piezoelectric coefficient and robust deposition process. Hereafter, AlScN is used synonymously with Al_0.73_Sc_0.27_N. AlN and AlScN films are deposited directly on (100) Si, SiO_2_ and poly-Si substrates, using process parameters established for the growth on metallic nucleation layers. The microstructure and *c*-axis texture quality of AlN and AlScN films are investigated using scanning electron microscopy (SEM), transmission electron microscopy (TEM), and high-resolution X-ray diffraction (XRD). The evaluation of the surface morphology and rocking curve XRD scans reveals that AlN films show a high degree of *c*-axis orientation on all investigated substrates. This confirms that sputter deposited AlN is able to realize a good texture with low distortions on many substrates, largely independent of the underlying texture [32,33,34,35,36]. In contrast, AlScN films grown directly on various nonmetallic substrates exhibit a high density of misaligned grains. The superior crystalline quality of AlN films motivates the approach reported herein to grow high quality AlScN on nonmetallic substrates using AlN as the nucleation layer.

## 2. Experimental

### 2.1. Sample Preparation

AlN and AlScN thin films are prepared by DC reactive magnetron sputtering in an Evatec Clusterline multichamber sputtering system. AlN films are sputter deposited in a gas plasma mixture of argon (Ar) and nitrogen (N_2_) at a temperature of 300 ${}^{\circ }$C, while AlScN films are prepared by cosputtering of Sc and Al targets in a pure N_2_ plasma system. The detailed process parameters are listed in Table 1. In this work, (100) Si, oxidized Si and poly-Si are used as substrates to grow AlN and AlScN films. All three substrates have a smooth surface on the front side (<2 nm RMS (root mean square)). Prior to the deposition of piezoelectric layers, all substrates are heated to 200 ${}^{\circ }$C for 30 s for degassing and then cleaned by a soft Ar plasma surface etching (300 W for 40 s) in vacuum. In the first part of this study, AlN as well as AlScN films with a thickness of 1 μm are deposited directly on three different substrates, respectively. In the second part, in order to investigate the effect of AlN seed layer on the crystalline orientation and piezoelectric response of AlScN thin films, a 20 nm AlN layer is first deposited on substrates, followed by a 500 nm AlScN layer after a vacuum break. For the characterization of the piezoelectric coefficient, the AlScN film has to be sandwiched between bottom and top electrodes [37]. Therefore, a stack of four layers consisting of bottom electrode, 20 nm AlN, 500 nm AlScN, and top electrode is deposited on an oxidized Si wafer. Here, 20 nm Ti/ 100 nm Pt and 200 nm Mo are used as bottom and top electrodes, respectively. All samples in this work are 8-inch wafer-scale in size.

### 2.2. Characterization Methods

The surface structure of AlN and AlScN thin films is investigated using a critical dimension scanning electron microscope (CDSEM, Hitachi), which allows loading and imaging of 8-inch wafers. The crystal structures of AlN and AlScN films are examined by performing XRD θ-2θ scans and ω scans in a Rigaku SmartLab diffractometer (9 kW, Hypix detector) with Cu Kα radiation, a Ge(220)x2 monochromator and soller slit of 5${}^{\circ }$.

Transmission electron microscopy analysis of the Si/AlN/AlScN stack is conducted on a cross-section specimen, which is prepared using the focused ion beam (FIB) method and milled down to electron-transparency (FEI DualBeam Helios600 FIB-SEM). The nanoscopic structural and chemical analyses are performed on a probe C_S_-corrected Titan^3^ G2 60-300 microscope operating at 300 kV and a JEOL JEM-2100 transmission electron microscope (thermionic source LaB_6_, acceleration voltage 200 kV) for selected area electron diffraction (SAED). The elemental distribution across the Si/AlN/AlScN interfaces is probed by energy dispersive X-ray spectroscopy (EDS) mapping using a Super-X EDS detector on the Titan^3^ microscope.

The piezoelectric coefficient $d_{33,f}$ is characterized using a double beam laser interferometer (DBLI) from aixACCT systems, which allows automatic measurement with an 8-inch wafer. As the piezoelectric coefficient depends on the ratio of the electrode size to the substrate thickness [38], the $d_{33,f}$ shown in this work has been calibrated to its geometry independent value.

## 3. Results and Discussion

### 3.1. Microstructure Investigations of AlN and AlScN Thin Films

**Figure 1:**
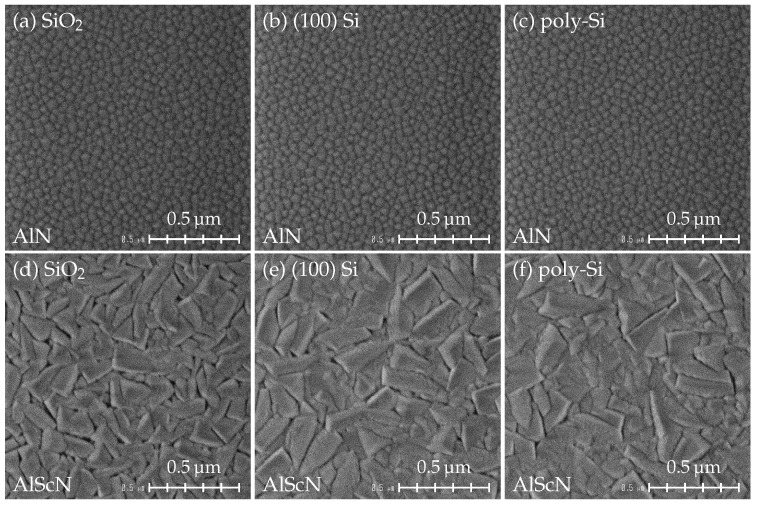
SEM surface view of 1 μm AlN deposited directly on (**a**) SiO_2_, (**b**) (100) Si, (**c**) poly-Si, and 1 μm AlScN deposited directly on (**d**) SiO_2_, (**e**) (100) Si, (**f**) poly-Si without a seed layer.without a seed layer.

Figure 1 shows the surface structures of AlN and AlScN films grown directly on three different substrates. All AlN samples (Figure 1a–c) show a homogeneous surface with small round grains, which indicates the successful growth of columnar grains with *c*-axis orientation as already reported in multiple studies [11,25,30]. On the other hand, the surface of AlScN on all three substrates (Figure 1d–f) is dominated by crystallites with wedge-shaped structure, implying a poor *c*-axis orientation [25]. In addition, AlScN films seem to be grown slightly better on Si and poly-Si substrates compared to SiO_2_ because few areas without misorientated grains can be observed.

**Figure 2:**
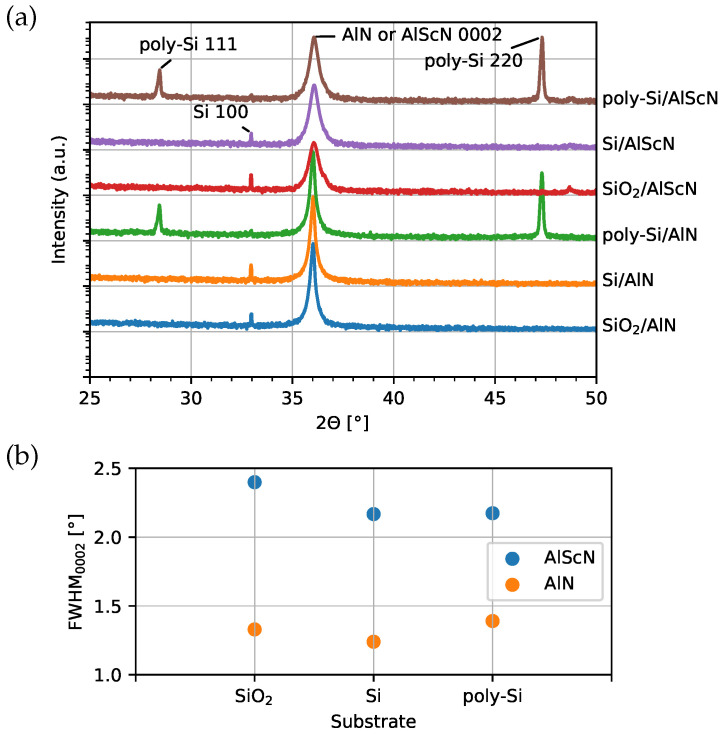
(**a**) θ-2θ scans of 1 μm AlN and AlScN grown directly on SiO_2_, Si and poly-Si substrates without a seed layer; (**b**) Results of rocking curve measurements of AlN and AlScN 0002 reflections. The FWHM is determined by fitting a pseudo-Voight profile using the XRD fit module (Python based open source tool for XRD peak fitting [42]).

To further examine the crystal phase and quality of the samples presented in Figure 1, XRD θ-2θ scans and ω scans are performed and shown in Figure 2. AlN and AlScN 0002 reflections at 2θ of around 36${}^{\circ }$ [39] are detected. In addition, the reflections of crystalline Si (100) orientation, poly-Si (111) and (220) planes are recorded in samples with corresponding substrates. The full width at half maximum (FWHM) of the AlN 0002 reflection rocking curve for all samples is less than 1.5${}^{\circ }$. This confirms that the AlN films on all investigated substrates are indeed well *c*-axis oriented. In contrast, for all AlScN samples, the measured FWHM values of 0002 reflection are larger than 2.2${}^{\circ }$, which is slightly higher than the reported FWHM of AlScN 0002 reflection (approx. 1.6${}^{\circ }$) reported in [25,39], which use Ti/Pt and Si as substrates, respectively. This indicates a lower quality of *c*-axis orientation of AlScN. The [0001] crystallographic direction of the out-of-the-plane misoriented grains has been shown to be tilted between 60${}^{\circ }$ and 90${}^{\circ }$ [40].

By using the given process parameters, the growth of highly *c*-axis oriented AlN directly on smooth surfaces (RMS < 2 nm) of amorphous SiO_2_, (100) Si and poly-Si wafers can be achieved, despite the different crystallographic texture of the substrate materials, in agreement with several studies [29,33,34,41]. However, a smooth surface alone is not sufficient to grow high quality AlScN films on these substrates as the misaligned grains and high FWHM values are measured. Our preliminary investigations show that the growth of AlScN on Ti/Pt bottom electrode for identical process parameters is stable and highly *c*-axis oriented (FWHM of 1.43${}^{\circ }$). Consequently, AlScN films are more sensitive to the substrate texture and irregularities. This fits with the observation that the *c*-axis orientation of AlScN even on metallic electrodes decreases significantly with increasing Sc concentration [25].

### 3.2. Microstructure and Piezoelectric Response of AlScN Films with a Thin AlN Seed Layer

To improve the *c*-axis orientation of AlScN on the investigated substrates, one option is to optimize the deposition parameters. In our previous work [43], we showed that the quality of Al_0.73_Sc_0.27_N films on SiO_2_ can be significantly improved by increasing the cathode–substrate distance offset. In this work, instead of optimizing the process parameters, an ultrathin AlN seed layer is introduced to improve the growth of *c*-axis oriented AlScN films on these substrates. We consider this approach to be more generally applicable and easier to transfer to different substrates.

**Figure 3:**
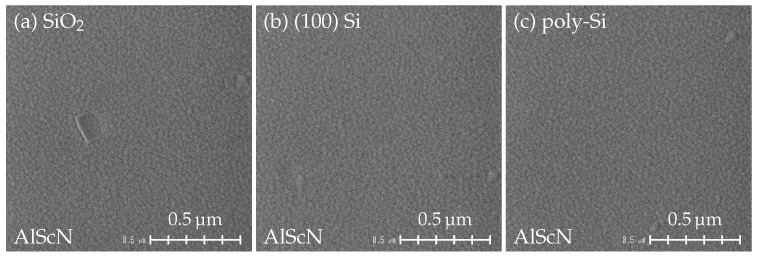
SEM surface view of 500 nm AlScN grown on (**a**) SiO_2_, (**b**) (100) Si and (**c**) poly-Si with a 20 nm AlN seed layer.

**Figure 4:**
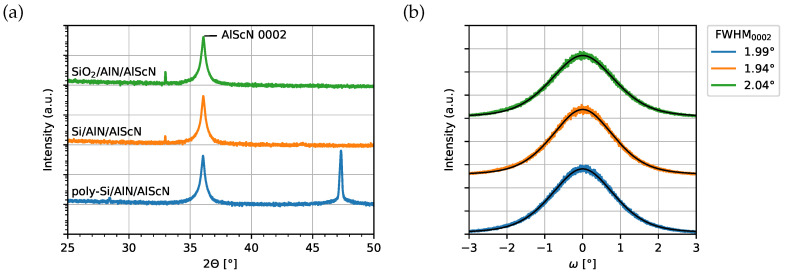
(**a**) θ-2θ scans of 500 nm AlScN grown on SiO_2_, Si and poly-Si substrates with the AlN seed layer; (**b**) Results of rocking curve measurements of AlScN 0002 reflections. The FWHM is determined by fitting a pseudo-Voight profile using the XRD fit module (Python-based open source tool for XRD peak fitting [42]).

The SEM images of 500 nm AlScN with 20 nm AlN seed layer on the investigated substrates are shown in Figure 3. Since the misoriented grains originate close to the substrate surface [25,30], there is no major difference in the number of misoriented grains between 500 nm and 1 μm AlScN films. A homogeneous surface with small grains is observed for all three samples, on which only a small number of misoriented grains is visible. Compared to the samples grown without the seed layer (Figure 1d–f), the *c*-axis orientation of AlScN films is significantly improved. The structural quality of AlN/AlScN films grown on SiO_2_, Si and poly-Si is characterized using XRD (Figure 4). The FWHM values of AlScN 0002 reflection are slightly below 2${}^{\circ }$ for all samples, which demonstrates only a moderate improvement in respect to Figure 1d–f. Although there is a small difference in 2θ values of AlN and AlScN 0002 orientations [10,11,39], a broadening of the 0002 FWHM due to the reflection from the 20 nm thin AlN seed layer can be expected to be negligible.

**Figure 5:**
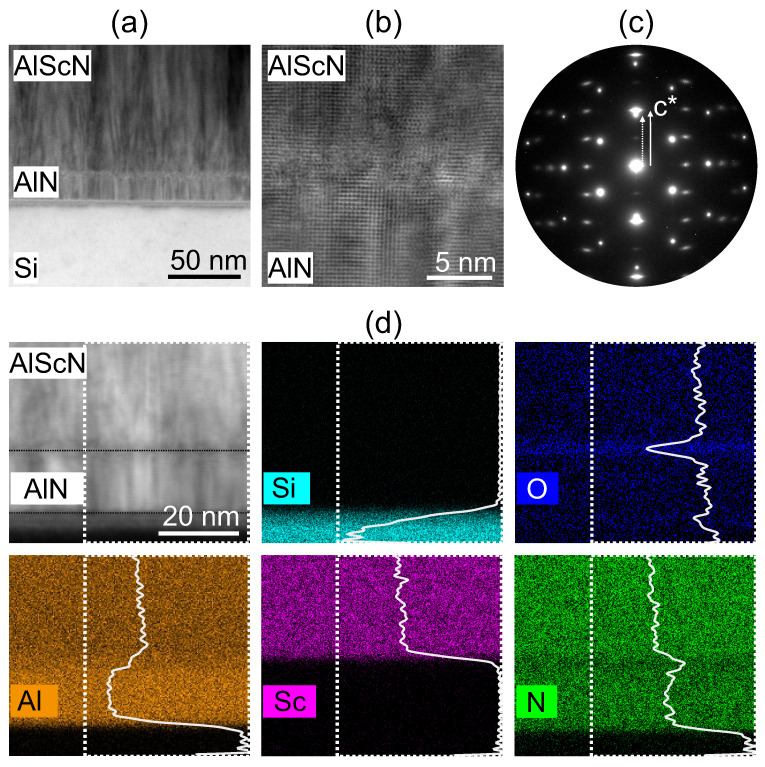
TEM study of the sample Si/AlN/AlScN. (**a**) STEM ABF overview image showing the columnar grain structures of AlN and AlScN layers on a natively passivated Si substrate; (**b**) HRSTEM ABF image showing structural disorder at the AlN/AlScN interface; (**c**) SAED pattern containing reflections of all layers corresponding to the [110] Si, [2$\bar{1}\bar{1}$0] and [1$\bar{1}$00] zone axes of AlN and AlScN; (**d**) STEM EDS elemental maps with integrated intensity profiles over the region of interest (dashed frame). The O-K map demonstrates the formation of an interfacial oxide layer between AlN and AlScN as well as the native oxide on the Si substrate.

For further investigation of the local chemical composition and nanostructure at the interfaces, the sample with Si/AlN/AlScN is selected for TEM analysis. The scanning TEM annular bright-field (ABF) micrograph in Figure 5a provides an overview of the film cross-section, showing columnar grain structures of AlN and AlScN layers. The quality of the AlN/AlScN interface is further investigated by high-resolution STEM imaging and elemental analysis. A magnified HRSTEM ABF image of the interface is given in Figure 5b. By using the ABF detector, contrast-rich images with atoms shown by black dots are recorded. The columnar grains with diameters < 5 nm growing along the *c*-axis on both sides of the interface are well displayed. However, obtaining a clear image of the interface is limited by the in-plane rotational disorder of the columnar grains and their three dimensional superposition along the finite sample thickness, as well as the patchy contrast spanning 2–3 nm in vertical direction across the interface region. The mosaic tilt along the *c*-axis is additionally visualized in the electron diffraction pattern recorded on the Si/AlN/AlScN multi-layers (see Figure 5c). The displayed intensity distribution can be explained by the superposition of the individual [110] Si, [2$\bar{1}\bar{1}$0] and [1$\bar{1}$00] AlN and AlScN zone axis patterns. The high coherency of the Si lattice causes electrons to scatter into sharp and bright reflections, whereas the different lattice constants of AlN and AlScN, as well as the small out-of-plane mosaic tilts of individual columns and the in-plane rotation of the fiber textured microstructure, result into diffuse and elongated intensities. The chemical composition is examined by elemental maps and profiles of the averaged intensity, as shown in Figure 5. Here, a peak in the oxygen signal is detected directly at the AlN/AlScN interface indicating a partial oxidation of the AlN surface during the vacuum break. Such partially oxidized interface has been reported before on a similar system and could not be avoided even after applying an RF etch cleaning step [44]. However, the oxide interface does not impede high-quality *c*-axis-oriented growth.

**Figure 6:**
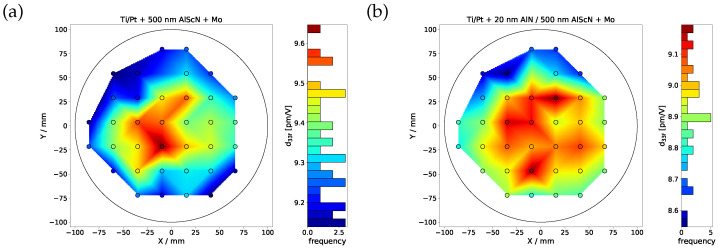
Measured $d_{33,f}$ of (**a**) 500 nm AlScN and of (**b**) 500 nm AlScN with 20 nm AlN seed layer on Ti/Pt_sput_ on a wafer level.

To investigate the effect of 20 nm AlN seed layer on the piezoelectric response, the piezoelectric coefficient d_33,f_ of 500 nm AlScN on sputtered Ti/Pt without and with the seed layer are measured and shown in Figure 6. The measured average d_33,f_ of AlScN layer with the seed layer is 8.91±0.03 pm/V, which is slightly lower (4.5%) compared to the one without the seed layer (9.33±0.02 pm/V). However, the homogeneity of the distribution is not affected. The slightly lower piezoelectric coefficient is due to the lower dielectric permittivity of AlN which limits the electric charge storage on electrode plates.

## 4. Conclusions

In this paper, we demonstrate a method to grow AlScN films with a high degree of *c*-axis orientation using same process parameters on various types of substrates, e.g., SiO_2_, (100) Si and poly-Si. This approach is to introduce a 20 nm thin AlN seed layer, which itself grows with good textural properties on most smooth substrates. By using an AlN seed layer, the wurtzite-type structure is established in the AlScN layer, resulting in a good *c*-axis orientation. The lattice mismatch between AlN and AlScN films seems to be of secondary importance in this context. In addition, there is only a small reduction (4.5%) from 9.33 pm/V to 8.91 pm/V in the piezoelectric coefficient d_33,f_ of AlScN layers in the presence of a 20 nm AlN seed layer with lower dielectric permittivity and piezoelectric coefficient.

## Figures and Tables

**Table 1:** Sputter deposition parameters of AlN and Al_0.73_Sc_0.27_N thin films.

	AlN	${\mathrm{Al}}_{0.73}{\mathrm{Sc}}_{0.27}N$
Power on Al target (kW)	7.5	4.5
Power on Sc target (kW)	/	3.5
Temperature (${}^{\circ }C$)	300	300
Ar flow (sccm)	28	/
N_2_ flow (sccm)	84	70

## Data Availability

The data presented in this study are available on reasonable request from the corresponding author.

## References

[1] Dubois M.A., Muralt P. (1999). Properties of aluminum nitride thin films for piezoelectric transducers and microwave filter applications. Appl. Phys. Lett..

[2] Kim T., Kim J., Dalmau R., Schlesser R., Preble E., Jiang X. (2015). High-temperature electromechanical characterization of AlN single crystals. IEEE Trans. Ultrason. Ferroelectr. Freq. Control.

[3] Jackson N., Keeney L., Mathewson A. (2013). Flexible-CMOS and biocompatible piezoelectric AlN material for MEMS applications. Smart Mater. Struct..

[4] Olivares J., Iborra E., Clement M., Vergara L., Sangrador J., Sanz-Hervás A. (2005). Piezoelectric actuation of microbridges using AlN. Sens. Actuators A Phys..

[5] Akiyama M., Kamohara T., Kano K., Teshigahara A., Takeuchi Y., Kawahara N. (2009). Enhancement of Piezoelectric Response in Scandium Aluminum Nitride Alloy Thin Films Prepared by Dual Reactive Cosputtering. Adv. Mater..

[6] Akiyama M., Kano K., Teshigahara A. (2009). Influence of growth temperature and scandium concentration on piezoelectric response of scandium aluminum nitride alloy thin films. Appl. Phys. Lett..

[7] Zywitzki O., Modes T., Barth S., Bartzsch H., Frach P. (2017). Effect of scandium content on structure and piezoelectric properties of AlScN films deposited by reactive pulse magnetron sputtering. Surf. Coat. Technol..

[8] Zukauskaite A., Wingqvist G., Palisaitis J., Jensen J., Persson P.O.A., Matloub R., Muralt P., Kim Y., Birch J., Hultman L. (2012). Microstructure and dielectric properties of piezoelectric magnetron sputtered w-Sc_x_Al_1-x_N thin films. J. Appl. Phys..

[9] Matloub R., Hadad M., Mazzalai A., Chidambaram N., Moulard G., Sandu C.S., Metzger T., Muralt P. (2013). Piezoelectric Al_1-x_Sc_x_N thin films: A semiconductor compatible solution for mechanical energy harvesting and sensors. Appl. Phys. Lett..

[10] Mertin S., Heinz B., Rattunde O., Christmann G., Dubois M.A., Nicolay S., Muralt P. (2018). Piezoelectric and structural properties of c-axis textured aluminium scandium nitride thin films up to high scandium content. Surf. Coat. Technol..

[11] Fichtner S., Reimer T., Chemnitz S., Lofink F., Wagner B. (2015). Stress controlled pulsed direct current co-sputtered Al_1-x_Sc_x_N as piezoelectric phase for micromechanical sensor applications. APL Mater..

[12] Su J., Niekiel F., Fichtner S., Thormaehlen L., Kirchhof C., Meyners D., Quandt E., Wagner B., Lofink F. (2020). AlScN-based MEMS magnetoelectric sensor. Appl. Phys. Lett..

[13] Yarar E., Fichtner S., Hayes P., Piorra A., Reimer T., Lisec T., Frank P., Wagner B., Lofink F., Meyners D. (2019). MEMS-Based AlScN Resonating Energy Harvester With Solidified Powder Magnet. J. Microelectromech. Syst..

[14] Gu-Stoppel S., Lisec T., Fichtner S., Funck N., Claus M., Wagner B., Lofink F. (2020). AlScN based MEMS quasi-static mirror matrix with large tilting angle and high linearity. Sens. Actuators A Phys..

[15] Matloub R., Artieda A., Sandu C., Milyutin E., Muralt P. (2011). Electromechanical properties of Al_0.9_Sc_0.1_N thin films evaluated at 2.5 GHz film bulk acoustic resonators. Appl. Phys. Lett..

[16] Park M., Hao Z., Dargis R., Clark A., Ansari A. (2020). Epitaxial Aluminum Scandium Nitride Super High Frequency Acoustic Resonators. J. Microelectromech. Syst..

[17] Kurz N., Ding A., Urban D.F., Lu Y., Kirste L., Feil N.M., Žukauskaitė A., Ambacher O. (2019). Experimental determination of the electro-acoustic properties of thin film AlScN using surface acoustic wave resonators. J. Appl. Phys..

[18] Gillinger M., Shaposhnikov K., Knobloch T., Schneider M., Kaltenbacher M., Schmid U. (2016). Impact of layer and substrate properties on the surface acoustic wave velocity in scandium doped aluminum nitride based SAW devices on sapphire. Appl. Phys. Lett..

[19] Schneider M., DeMiguel-Ramos M., Flewitt A.J., Iborra E., Schmid U. (2017). Scandium Aluminium Nitride-Based Film Bulk Acoustic Resonators. Proceedings.

[20] Wang J., Park M., Mertin S., Pensala T., Ayazi F., Ansari A. (2020). A Film Bulk Acoustic Resonator Based on Ferroelectric Aluminum Scandium Nitride Films. J. Microelectromech. Syst..

[21] Meyer J.M., Schell V., Su J., Fichtner S., Yarar E., Niekiel F., Giese T., Kittmann A., Thormählen L., Lebedev V. (2021). Thin-Film-Based SAW Magnetic Field Sensors. Sensors.

[22] Jackson N. (2016). Influence of silicon crystal orientation on piezoelectric textured aluminium nitride deposited on metal electrodes. Vacuum.

[23] Felmetsger V.V., Laptev P.N., Tanner S.M. Crystal orientation and stress in AC reactively sputtered AlN films on Mo electrodes for electro-acoustic devices. Proceedings of the 2008 IEEE Ultrasonics Symposium.

[24] Lee J.B., Jung J.P., Lee M.H., Park J.S. (2004). Effects of bottom electrodes on the orientation of AlN films and the frequency responses of resonators in AlN-based FBARs. Thin Solid Film..

[25] Fichtner S., Wolff N., Krishnamurthy G., Petraru A., Bohse S., Lofink F., Chemnitz S., Kohlstedt H., Kienle L., Wagner B. (2017). Identifying and overcoming the interface originating c-axis instability in highly Sc enhanced AlN for piezoelectric micro-electromechanical systems. J. Appl. Phys..

[26] Kamohara T., Akiyama M., Ueno N., Kuwano N. (2008). Improvement in crystal orientation of AlN thin films prepared on Mo electrodes using AlN interlayers. Ceram. Int..

[27] Alvarado M., Pelegrini M., Pereyra I., Assumpção T.d., Kassab L., Alayo M. (2014). Fabrication and characterization of aluminum nitride pedestal-type optical waveguide. Can. J. Phys..

[28] Meinel K., Melzer M., Stoeckel C., Shaporin A., Forke R., Zimmermann S., Hiller K., Otto T., Kuhn H. (2020). 2D Scanning Micromirror with Large Scan Angle and Monolithically Integrated Angle Sensors Based on Piezoelectric Thin Film Aluminum Nitride. Sensors.

[29] Jiao X., Shi Y., Zhong H., Zhang R., Yang J. (2015). AlN thin films deposited on different Si-based substrates through RF magnetron sputtering. J. Mater. Sci. Mater. Electron..

[30] Lu Y., Reusch M., Kurz N., Ding A., Christoph T., Kirste L., Lebedev V., Žukauskaitė A. (2018). Surface Morphology and Microstructure of Pulsed DC Magnetron Sputtered Piezoelectric AlN and AlScN Thin Films. Phys. Status Solidi A.

[31] Teshigahara A., Hashimoto K.Y., Akiyama M. (2012). Scandium aluminum nitride: Highly piezoelectric thin film for RF SAW devices in multi GHz range. Proceedings of the 2012 IEEE International Ultrasonics Symposium.

[32] Gillinger M., Shaposhnikov K., Knobloch T., Stöger-Pollach M., Artner W., Hradil K., Schneider M., Kaltenbacher M., Schmid U. (2018). Enhanced c-axis orientation of aluminum nitride thin films by plasma-based pre-conditioning of sapphire substrates for SAW applications. Appl. Surf. Sci..

[33] Iriarte G., Rodríguez J., Calle F. (2010). Synthesis of c-axis oriented AlN thin films on different substrates: A review. Mater. Res. Bull..

[34] Artieda A., Sandu C., Muralt P. (2010). Highly piezoelectric AlN thin films grown on amorphous, insulating substrates. J. Vac. Sci. Technol. A Vac. Surfaces Film..

[35] Clement M., Vergara L., Sangrador J., Iborra E., Sanz-Hervás A. (2004). SAW characteristics of AlN films sputtered on silicon substrates. Ultrasonics.

[36] Xiong J., Gu H., Hu K.I., Hu M.Z. (2010). Influence of substrate metals on the crystal growth of AlN films. Int. J. Miner. Metall. Mater..

[37] Liu J.M., Pan B., Chan H., Zhu S., Zhu Y., Liu Z. (2002). Piezoelectric coefficient measurement of piezoelectric thin films: An overview. Mater. Chem. Phys..

[38] Sivaramakrishnan S., Mardilovich P., Schmitz-Kempen T., Tiedke S. (2018). Concurrent wafer-level measurement of longitudinal and transverse effective piezoelectric coefficients (d_33,f_ and e_31,f_) by double beam laser interferometry. J. Appl. Phys..

[39] Lu Y., Reusch M., Kurz N., Ding A., Christoph T., Prescher M., Kirste L., Ambacher O., Zukauskaite A. (2018). Elastic modulus and coefficient of thermal expansion of piezoelectric Al_1-x_Sc_x_N (up to x = 0.41) thin films. APL Mater..

[40] Sandu C.S., Parsapour F., Mertin S., Pashchenko V., Matloub R., LaGrange T., Heinz B., Muralt P. (2019). Abnormal Grain Growth in AlScN Thin Films Induced by Complexion Formation at Crystallite Interfaces. Phys. Status Solidi A.

[41] Tamariz S., Martin D., Grandjean N. (2017). AlN grown on Si (111) by ammonia-molecular beam epitaxy in the 900–1200 ^∘^C temperature range. J. Cryst. Growth.

[42] Crowther P., Daniel C.S. (2020). xrdfit: A Python package for fitting synchrotron X-ray diffraction spectra. J. Open Source Softw..

[43] Fichtner S. (2020). Development of High Performance Piezoelectric AlScN for Microelectromechanical Systems: Towards a Ferroelectric Wurtzite Structure.

[44] Parsapour F., Pashchenko V., Mertin S., Sandu C., Kurz N., Nicolay P., Muralt P. Ex-situ AlN seed layer for (0001)-textured Al_0.84_Sc_0.16_N thin films grown on SiO_2_ substrates. Proceedings of the 2017 IEEE International Ultrasonics Symposium (IUS).

